# Abolishment of morphology-based taxa and change to binomial species names: 2022 taxonomy update of the ICTV bacterial viruses subcommittee

**DOI:** 10.1007/s00705-022-05694-2

**Published:** 2023-01-23

**Authors:** Dann Turner, Andrey N. Shkoporov, Cédric Lood, Andrew D. Millard, Bas E. Dutilh, Poliane Alfenas-Zerbini, Leonardo J. van Zyl, Ramy K. Aziz, Hanna M. Oksanen, Minna M. Poranen, Andrew M. Kropinski, Jakub Barylski, J Rodney Brister, Nina Chanisvili, Rob A. Edwards, François Enault, Annika Gillis, Petar Knezevic, Mart Krupovic, Ipek Kurtböke, Alla Kushkina, Rob Lavigne, Susan Lehman, Malgorzata Lobocka, Cristina Moraru, Andrea Moreno Switt, Vera Morozova, Jesca Nakavuma, Alejandro Reyes Muñoz, Jānis Rūmnieks, BL Sarkar, Matthew B. Sullivan, Jumpei Uchiyama, Johannes Wittmann, Tong Yigang, Evelien M. Adriaenssens

**Affiliations:** 1 School of Applied Sciences, College of Health, Science and Society, University of the West of England, Bristol, BS16 1QY UK; 2 Department of Medicine and APC Microbiome Ireland, School of Microbiology, University College Cork, Cork, Ireland; 3 Department of Biosystems, Faculty of Bioscience Engineering, KU, Leuven, Belgium; 4 Department of Genetics and Genome Biology, University of Leicester, University Road, Leicester, UK; 5 Institute of Biodiversity, Faculty of Biological Sciences, Cluster of Excellence Balance of the Microverse, Friedrich-Schiller-University Jena, 07743 Jena, Germany; 6 Theoretical Biology and Bioinformatics, Science for Life, Utrecht University, Padualaan 8, Utrecht, 3584 CH The Netherlands; 7 Departamento de Microbiologia, Universidade Federal de Viçosa, Viçosa, MG 36570-900 Brazil; 8 Institute for Microbial Biotechnology and Metagenomics (IMBM), Department of Biotechnology, University of the Western Cape, 7535 Bellville, Cape Town, South Africa; 9 Department of Microbiology and Immunology, Faculty of Pharmacy, Cairo University, 11562 Cairo, Egypt; 10 Egypt/ and Children’s Cancer Hospital, 57357, 11617 Cairo, Egypt; 11 Molecular and Integrative Biosciences Research Programme, Faculty of Biological and Environmental Sciences, University of Helsinki, Viikinkaari 9, 00014 Helsinki, Finland; 12 Department of Pathobiology, Ontario Veterinary College, University of Guelph, Guelph, ON N1G 2W1 Canada; 13 Department of Molecular Virology, Adam Mickiewicz University in Poznan, Poznan, Poland; 14 National Center for Biotechnology Information, National Library of Medicine, National Institutes of Health, Bethesda, MD 20894 USA; 15 The Eliava Institute of Bacteriophage, Microbiology and Virology, Tbilisi, Georgia; 16 Flinders Accelerator for Microbiome Exploration, Adelaide, Australia; 17 Université Clermont Auvergne, CNRS, LMGE, Clermont-Ferrand, France; 18 Laboratory of Food and Environmental Microbiology, Université Catholique de Louvain, Croix du Sud 2, L7.05.12, 1348 Louvain-la-Neuve, Belgium; 19 PK Lab, Department of Biology and Ecology, Faculty of Sciences, University of Novi Sad, Trg Dositeja Obradovica 3, Novi Sad, Serbia; 20 Archaeal Virology Unit, Institut Pasteur, Université Paris Cité, CNRS UMR6047, Paris, 75015 France; 21 School of Science, Technology and Engineering, University of the Sunshine Coast, 4558 Maroochydore, BC, QLD Australia; 22 Department of Bacteriophage molecular genetics, D.K.Zabolotny Institute of microbiology and virology, NAS of Ukraine, 154 Acad. Zabolotnoho str, 03143 Kyiv, Ukraine; 23 Department of Bacterial molecular genetics, Faculty of biology, University of Gdansk, Wita Stwosza 59, 80-308 Gdansk, Poland; 24 Center for Biologics Evaluation and Research, Food and Drug Administration, Silver Spring, MD USA; 25 Institute of Biochemistry and Biophysics, Polish Academy of Sciences, Warsaw, Poland; 26 Institute for Chemistry and Biology of the Marine Environment, University of Oldenburg, Oldenburg, Germany; 27 Escuela de Medicina Veterinaria, Facultad de Agronomía e Ingeniería Forestal, Facultad de Ciencias Biológicas y Facultad de Medicina, Pontificia Universidad Católica de Chile, Santiago, Chile; 28 Laboratory of Molecular Microbiology, Institute of Chemical Biology and Fundamental Medicine SB RAS, Novosibirsk, Russia; 29 College of Veterinary Medicine, Animal Resources and Biosecurity, Makerere University, P. O. Box 7062, Kampala, Uganda; 30 Max Planck Tandem Group in Computational Biology, Departamento de Ciencias Biológicas, Universidad de los Andes, 111711 Bogotá, Colombia; 31 Latvian Biomedical Research and Study Center, 1067 Riga, Latvia; 32 ICMR-National Institute of Cholera and Enteric Diseases (NICED), Kolkata, India; 33 Departments of Microbiology and Civil, Environmental, and Geodetic Engineering, Ohio State University, Columbus, OH 43210 USA; 34 Department of Bacteriology, Graduate School of Medicine Dentistry and Pharmaceutical Sciences, Okayama University, 1-1-1, Tsushima-naka, Kita-ku, Okayama, 7008530 Japan; 35 Leibniz Institute DSMZ - German Collection of Microorganisms and Cell Cultures GmbH, Inhoffenstr. 7B, 38124 Braunschweig, Germany; 36 College of Life Science and Technology, Beijing University of Chemical Technology, Beijing, 100029 China; 37 Quadram Institute Bioscience, Rosalind Franklin Road, Norwich Research Park, Norwich, NR4 7UQ UK

## Abstract

This article summarises the activities of the Bacterial Viruses Subcommittee of the International Committee on Taxonomy of Viruses for the period of March 2021−March 2022. We provide an overview of the new taxa proposed in 2021, approved by the Executive Committee, and ratified by vote in 2022. Significant changes to the taxonomy of bacterial viruses were introduced: the paraphyletic morphological families *Podoviridae*, *Siphoviridae*, and *Myoviridae* as well as the order *Caudovirales* were abolished, and a binomial system of nomenclature for species was established. In addition, one order, 22 families, 30 subfamilies, 321 genera, and 862 species were newly created, promoted, or moved.

**Supplementary Information:**

The online version contains supplementary material available at 10.1007/s00705-022-05694-2.

## Introduction

Here, we present taxonomic changes submitted to the Bacterial Viruses Subcommittee (BVS) of the International Committee on Taxonomy of Viruses (ICTV) in 2021, assessed and approved at the ICTV Executive Committee meeting (EC53, July 2021), and ratified in 2022 by membership vote [1]. The new taxonomy release (#37) can be found on the ICTV website (https://ictv.global/) and is available for download as two Excel files, the Master Species List (MSL37) and the Virus Metadata Resource (VMR37), which links each species with an exemplar virus genome and accession number.

### Abolishment of the families *Myoviridae*, *Podoviridae*, and *Siphoviridae* and the order *Caudovirales*

The most drastic change in phage classification officialised with this release is the abolishment of the morphology-based families *Myoviridae*, *Podoviridae*, and *Siphoviridae* and the removal of the order *Caudovirales*, which is replaced by the class *Caudoviricetes* to group all tailed bacterial and archaeal viruses with icosahedral capsids and a double-stranded DNA genome. The submission of the taxonomy proposal (2021.001B) followed the publication of a manuscript describing a roadmap for phage taxonomy by the Vice Chair, former Chair, and current Chair of the BVS [2]. This change was necessary, given multiple independent assessments that these morphology-based families are polyphyletic and do not accurately reflect shared evolutionary histories [3–8].

The process of accommodating all members of the three morphology-based families into new, genomically coherent families has begun. Creating new families requires at least two genera, but ideally more, to enable definition of demarcation criteria based on core gene sets and phylogenetic analysis. While this leaves some taxa as “unclassified” at the levels of family and order within the class *Caudoviricetes* (summarised in Supplementary Table S1), it allows for the creation of new genome-based taxa that better reflect the diversity and genomic relationships of these abundant and diverse viruses in the future. At the same time, we recognise the importance of morphological (non-taxonomic) identifiers such as "podovirus", "myovirus", or "siphovirus". These terms can be used freely to reflect these distinctive features and retain their historical reference; however, after the 2022 ratification vote, they do not have any formal taxonomic meaning/significance.

## Change to freeform binomial species name format

In 2020, the ICTV voted to mandate the binomial format for the naming of virus species, where the genus name and a species epithet together form a unique species name, consistent with other taxonomies in biology [9, 10]. This change was applied to 2,532 species of bacterial viruses with input from each study group chair and the members of the BVS through an online consultation. The BVS elected to implement a fully freeform format for binomial naming in which the study groups and taxonomy proposal authors were responsible for the final format of the species names. A brief description of how this binomial format was applied to bacterial viruses follows.

In most cases, the species epithet was directly inherited from the exemplar virus name. For example, the species *Escherichia virus T4* was renamed to *Tequatrovirus T4*, reflecting the name of its exemplar phage T4 (also called Enterobacteria phage T4, phage T4, or Escherichia phage T4). Certain study groups and proposal authors elected to define new species epithets, such as the *Leviviricetes* and *Crassvirales* Study Groups, who proposed latinised binomials for all species. For example, the model phage MS2 was assigned to the species *Emesvirus zinderi* [11], the prototypical crAssphage was assigned to the species *Carjivirus communis*, and the first isolated representative of the order *Crassvirales*, Bacteroides phage crAss001, was assigned to the species *Kehishuvirus primarius* [12].

The BVS implemented a number of additional orthographic rules for the creation of new species epithets that are derived directly from the phage name. Where virus names consisted only of numerals, the first letter of the genus names, followed by a “v” (virus) was prepended to the species epithet. For example, Pseudomonas phage 14 − 1 is the exemplar virus of species *Pbunavirus pv141*. The species epithet can be composed of a mixture of alphanumeric characters in lower and upper case. For future species epithets, we would request that only lowercase letters are used. Where existing phage names had a mixture of upper and lower case letters, these were retained to facilitate recognition of similar virus names such as P2 and p2 by the bacterial virus research community.

Where the species epithet consisted of letters only, the consensus of the BVS was to only permit lowercase letters. For example, Bacillus phage Deep Blue is the exemplar virus of the species *Caeruleovirus deepblue*.

## Overview of taxonomy changes

Ninety-five taxonomy proposals were submitted to the BVS Chair and approved by the Executive Committee in 2021. The number of proposals and taxa created precludes detailed individual descriptions, but they are summarised in Supplementary Table S1, and all individual proposals are available from the ICTV website (https://ictv.global/files/proposals/approved?fid=4467#block-teamplus-page-title). Herein, we provide an overview of changes (Table 1) and a brief summary of proposals resulting in the creation of higher-ranking taxa, including new orders and families.

**Table 1:** Summary of taxonomic changes for bacterial viruses for Master Species List 37, ratified in 2022. No changes were made at the class, phylum, kingdom, or realm level.

	Species	Genus	Subfamily	Family	Order
Abolished	5	2	0	3	1
New	804	257	26	21	1
Moved or promoted	58	64	4	1	0
Renamed	2736	1	0	0	0
Current total	3601	1199	98	47	4

**Fig. 1:**
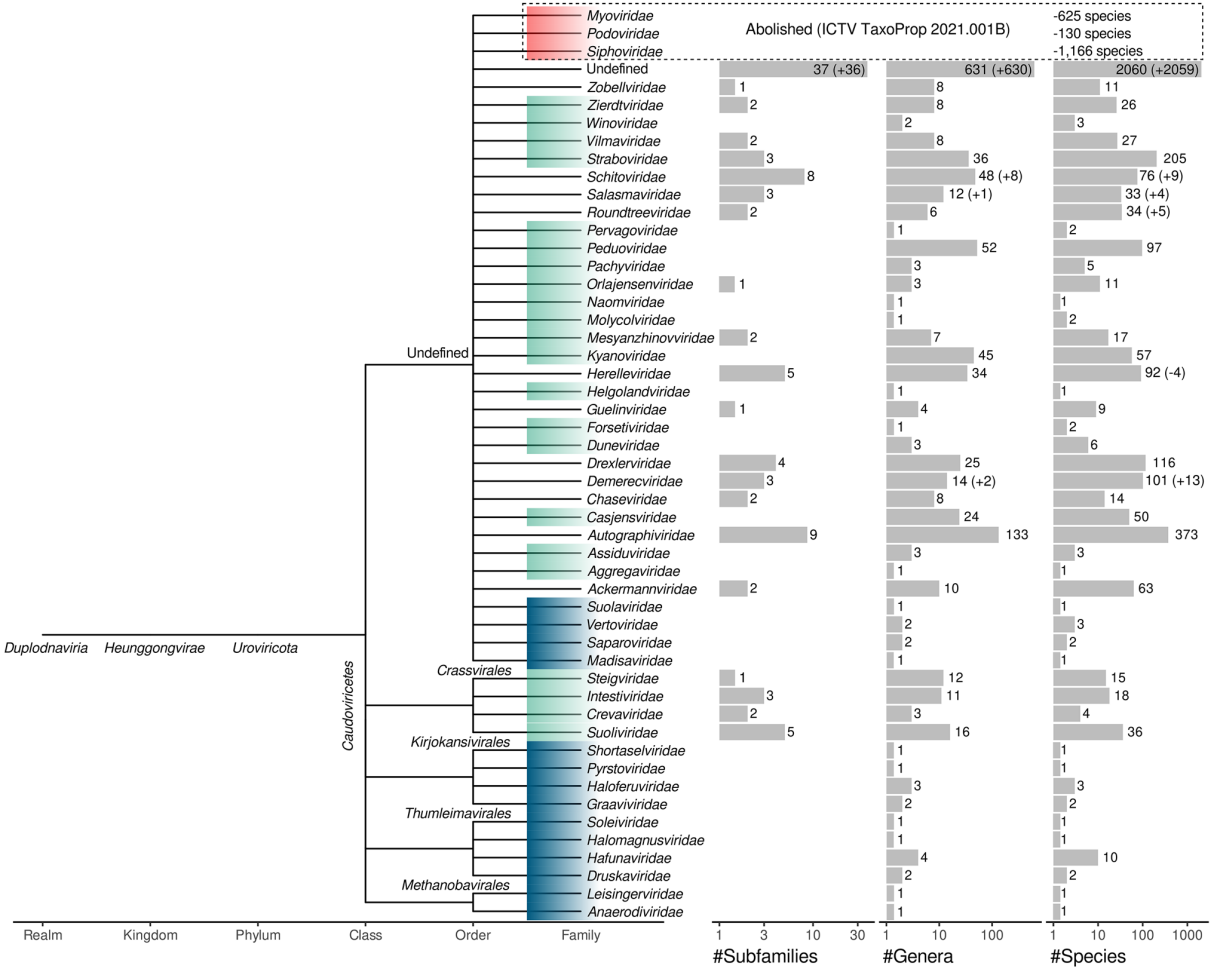
Cladogram depicting the taxonomic structure of the order *Caudoviricetes*. New bacterial and archaeal virus families created in 2021 are highlighted in green or blue, respectively, and those abolished are in red. Numbers of subfamilies, genera, and species are depicted as histograms adjacent to each family. Numbers in parentheses indicate changes recorded from ratified taxonomic proposals. Subfamilies and genera moved from the abolished families *Myoviridae*, *Podoviridae*, and *Siphoviridae* are detailed in Supplementary Table S1.

The class *Caudoviricetes* now contains 14 families assigned to four orders. Three of these orders encompass viruses infecting archaea [1, 13]. A further 33 families have been established but not yet been assigned to an order, in addition to 37 subfamilies and 631 genera that have yet to be classified at the rank of family or order (Fig. 1).

### New taxa in the class *Caudoviricetes*

Twenty-two new families were delineated in the class *Caudoviricetes*, 21 of which were newly established (four within the new order *Crassvirales*) and one of which was promoted from the level of subfamily (Table 1). Additional taxa (Fig. 1) were established in the class *Caudoviricetes* by the Archaeal Viruses Subcommittee [1, 13] but are not discussed in this report.

### Order *Crassvirales*

The newly established order *Crassvirales*, named after the computer program crAss [14], which was used in the discovery of its founding member, comprises dsDNA tailed bacteriophages with genome sizes ranging from 83 to 106 kbp (Taxonomy Proposal 2021.021B.A.Crassvirales). Very few representatives of the order have been isolated in culture. The majority of species have been described based on analysis of metagenomically assembled genomes (MAGs) extracted from gut-associated, soil, marine, and industrial environments [15–18]. Members of the order *Crassvirales* are especially prevalent in mammalian gut microbiomes and were isolated from, or predicted to infect, bacteria of the phylum Bacteroidota (more specifically, order Bacteroidales in the mammalian gut) [19–21].

The first representative to be discovered and reported in the literature was crAssphage, a ubiquitous and highly prevalent member of the human gut virome [19, 22]. Metagenomic sequencing reads mapped to the prototypical crAssphage from up to 73% of the human faecal metagenomic datasets, accounting for up to 90% of the reads in virus-like-particle-enriched metagenomes or 22% of the reads of the total metagenomes in extreme cases. With other members of the order included, these viruses are detectable in 98% of faecal viromes from Western cohorts and in 77% of human faecal viromes worldwide. In 8% of cases, crAss-like phages accounted for > 50% of the virome, making this order the most abundant group of viruses of the human virome [16].

The current genome-based taxonomy of the order *Crassvirales* includes four families (*Intestiviridae*, *Crevaviridae*, *Suoliviridae*, and *Steigviridae*), 11 subfamilies, 42 genera, and 73 species. Cultured representatives only exist for the families *Intestiviridae* (species *Jahgtovirus secundus* represented by Bacteroides phage crAss002) and *Steigviridae* (species *Kehishuvirus primarius* represented by Bacteroides phage crAss001, *Wulfhauvirus bangladeshii* by Bacteroides phages DAC15 and DAC17, and *Akihdevirus balticus* by Cellulophaga phage phi14:2). *Carjivirus communis*, the species comprising the prototypical crAssphage, currently remains without cultured isolates.

### Family *Peduoviridae*

The family *Peduoviridae* is the formalisation of P2-like phages consisting of tailed, non-enveloped viruses that contain a set of six orthologous core genes. This group of viruses was previously a subfamily (*Peduovirinae*) in the abolished family *Myoviridae* but warranted elevation to family status when the morphology-based classification of viruses was abolished and genomic analysis revealed that the diversity among members of the *Peduovirinae* (created in 2011; Taxonomy Proposal 2009.012a-qB) is similar to that of other recently defined families in the class *Caudoviricetes*. The family includes arguably the best-studied example of a class of temperate phage commonly found in genomes of Gram-negative bacteria, P2 (isolated in 1951 by Giuseppe Bertani [23]), after which the family is named.

### Family *Mesyanzhinovviridae*

The family *Mesyanzhinovviridae* is named in honour of the late Vadim V. Mesyanzhinov (Shemyakin-Ovchinnikov Institute Moscow). The family encompasses seven genera comprising temperate and lytic siphoviruses infecting strains of the eubacterial genera *Bordetella, Pseudomonas*, and *Xanthomonas*. It consists of two subfamilies – *Bradleyvirinae*, named for David Edward Bradley, known for his research into the ultrastructure of bacteriophages (genera: *Abidjanvirus, Bosavirus, Xooduovirus*, and *Epaquintavirus*) and *Rabinowitzvirinae*, named for Genia Rabinowitz, one of the first scientists to study bacteriophages of *Bacillus pyocyaneus* sp. (genera: *Vojvodinavirus* and *Yuavirus*) – and the orphan genus *Keylargovirus*.

### Families *Straboviridae* and *Kyanoviridae*

The family *Straboviridae* is named after the Greek philosopher Strabo. The new family encompasses the previous “T4-like” phages classified within the abolished family *Myoviridae*. The family *Straboviridae* now consists of 35 genera, with 11 newly created genera (genera: *Kagamiyamavirus, Angelvirus, Bragavirus, Mosugukvirus, Mylasvirus, Roskildevirus, Jiangsuvirus, Chrysonvirus, Cinqassovirus*, *Gualtarvirus*, and *Carettavirus*). There are 21 genera within the subfamilies *Tevenvirinae* (genera: *Dhakavirus, Gaprivervirus, Gelderlandvirus, Jiaodavirus, Kagamiyamavirus, Kanagawavirus, Karamvirus, Moonvirus, Mosigvirus, Mosugukvirus, Roskildevirus, Tegunavirus, Tequatrovirus*, and *Winklervirus), Emmerichvirinae* (genera: *Ishigurovirus* and *Ceceduovirus*), and *Twarogvirinae* (genera: *Lasallevirus*, *Hadassahvirus, Acajnonavirus*, and *Zedzedvirus*), with a further 14 floating genera (*Angelvirus*, *Biquartavirus*, *Bragavirus*, *Carettavirus*, *Chrysonvirus*, *Cinqassovirus, Gualtarvirus, Jiangsuvirus, Krischvirus*, *Mylasvirus*, *Pseudotevenvirus*, *Schizotequatrovirus*, *Slopekvirus*, and *Tulanevirus).* The genera S*chizotequatrovirus, Slopekvirus, Pseudotevenvirus*, and *Krischvirus* have been removed from the subfamily *Tevenvirinae*, as the members of these genera are not monophyletic with other members of the subfamily.

The family *Kyanoviridae* is named after the ancient Greek word *Kyanos* for “cyan/dark blue”. The family includes “T4-like” phages of the myovirus morphotype, with all representatives to date infecting cyanobacteria. While related to members of the *Straboviridae*, they are distant relatives. As such, and to be consistent with other viral families, they have been separated into a family, as they form a monophyletic group. The relationship between *Straboviridae*, *Kyanoviridae*, and other families at higher taxonomic levels has yet to be resolved. Currently there are no subfamilies, and the family consists of 26 genera previously of the abolished *Myoviridae* (*Cymopoleiavirus*, *Charybdisvirus*, *Anaposvirus*, *Nodensvirus*, *Vellamovirus*, *Kanaloavirus*, *Bellamyvirus*, *Salacisavirus*, *Mazuvirus*, *Atlauavirus*, *Libanvirus*, *Ahtivirus*, *Namakavirus*, *Pontusvirus*, *Ronodorvirus*, *Leucotheavirus*, *Thetisvirus*, *Thaumasvirus*, *Tefnutvirus*, *Neptunevirus*, *Palaemonvirus*, *Nerrivikvirus*, *Brizovirus*, *Nereusvirus*, *Acionnavirus*, and *Biquartavirus*) and 20 new genera (*Sedonavirus*, *Gibbetvirus*, *Chalconvirus*, *Potamoivirus*, *Lipsvirus*, *Lowelvirus*, *Makelovirus*, *Haifavirus*, *Nilusvirus*, *Sokavirus*, *Macariavirus*, *Greenvirus*, *Ormenosvirus*, *Shandvirus*, *Emcearvirus, Galenevirus*, *Glaucusvirus*, *Alisovirus*, *Neritesvirus*, and *Bristolvirus)*.

### **Family***Naomviridae*

The family *Naomviridae* consists of the single genus *Noahvirus*. The etymology of the family name comes from the Hebrew meaning of Naomi, “pleasant one/good one”. Current members of the genus *Noahvirus* have the defining characteristic of replacing deoxythymidine with deoxyuridine within their genomic DNA [24].

### Family *Casjensviridae*

The family *Casjensviridae*, named in honour of Sherwood R. Casjens (University of Utah), comprises the four existing genera (*Ahduovirus, Chivirus, Nazgulvirus* and *Sanovirus*) and 20 new genera (*Broinstvirus, Cenphatecvirus, Dunedinvirus, Enchivirus, Fengtaivirus, Gediminasvirus, Gwanakrovirus, Jacunavirus, Kokobelvirus, Lavrentievavirus, Maxdohrnvirus, Newforgelanevirus, Phobosvirus, Redjacvirus, Salvovirus, Seodaemunguvirus, Sharonstreetvirus, Yonseivirus*, and *Zhonglingvirus*) of flagellotrophic siphoviruses, including Salmonella phage Chi (assigned to the species *Chivirus chi*) [25].

### Family *Vilmaviridae*

The family *Vilmaviridae* was named after clusters V, L, and M, defined in the Actinobacteriophage Database [26]. The family is composed of two subfamilies, *Lclasvirinae* (genera: *Bromdenvirus*, *Bronvirus*, *Faithunavirus*, and *Lumosvirus*) and *Mclasvirinae* (genera: *Bongovirus* and *Reyvirus*) and two unassigned genera, *Kumaovirus* and *Wildcatvirus*. The L and M cluster viruses are all temperate siphoviruses, while those belonging to cluster V are strictly lytic *Mycobacterium* siphophages.

### Family *Orlajensenviridae*

The family *Orlajensenviridae* formalises the classification of the cluster EE *Microbacterium* phages defined in the Actinobacteriophage Database [26]. The family is composed of a single subfamily, *Pelczarvirinae*, named after Michael Joseph Pelczar, which includes three genera: *Paopuvirus*, *Bonaevitaevirus*, and *Efekovirus*, which share 20 orthologous core genes encoding structural, morphogenesis, and DNA-binding proteins. The family is named after Sigurd Ola-Jensen, who was responsible for the initial isolation and taxonomy of the bacterial genus *Microbacterium*.

### Bacterial viruses infecting members of the bacterial class Flavobacteriia are classified within nine families

Nine new families were established, based on comparative and phylogenetic analysis of isolated bacteriophages infecting members of the bacterial class Flavobacteriia and supported by complete coding sequences from metagenomes. Each of the families forms a monophyletic group in VirClust [27] and VICTOR [28], and their members share at least eight core genes [28]. Members of these families infect bacterial hosts of the genera *Polaribacter*, *Tenacibaculum*, *Cellulophaga*, *Winogradskyella*, and *Olleya*.

Two families are named after the islands Helgoland and Dune of the Helgoland Archipelago in the North Sea, where many of these bacterial viruses were isolated: *Helgolandviridae*, which contains a single genus (*Leefvirus*), and *Duneviridae*, which contains three genera (*Ingelinevirus Labanvirus* and *Unahavirus*). The family *Forsetiviridae* is named after the god Forseti, associated with the island Hegoland in Nordic mythology, and consists of a single genus, *Freyavirus*. Derived from the Latin word *pervagus*, translated as “widely roaming”, the family *Pervagoviridae* is composed of a single genus, *Callevirus*. *Pachyviridae*, named after the Greek word *pakhús* for “thick”, consists of three genera (*Baltivirus*, *Bacelvirus*, and *Gundelvirus*). *Molycolviridae* is named after the two species in the genus *Mollyvirus*: *Mollyvirus molly and Mollyvirus colly*. Three genera (*Nekkelsvirus*, *Cebadecemvirus*, and *Cellubavirus*) are classified within the family *Assiduviridae*, named after the Latin word *assiduous*, meaning “constant” or “regular”. The family *Aggregaviridae*, so named because the host *Olleya* sp. are commonly found on aggregates, consists of a single genus, *Harrekavirus*. Lastly, the family *Winoviridae* is named after the host genus *Winogradskyella* and contains two genera, *Peternellavirus* and *Pippivirus*.

### **Family***Zierdtviridae*

The family *Zierdtviridae*, named after Charles Henry Zierdt, contains two subfamilies: *Emilbogenvirinae* (genera: *Sukkupivirus*, *Kablunavirus*, *Foxborovirus*, *Pleakleyvirus*, *Skysandvirus*, and *Gruunavirus*) and *Toshachvirinae* (genera: *Chunghsingvirus* and *Ceetrepovirus*), comprising lytic *Gordonia* phages from cluster CR and *Corynebacterium* phages of cluster EN defined in the Actinobacteriophage Database [26]. The subfamilies were named after Sheila Toshach and Emil Bogen. Members of the family share a set of 18 core genes.

## Conclusions and future directions

The past year has seen substantial changes in the taxonomy of bacterial viruses, as the BVS and ICTV move towards a more coherent and unified system of virus classification based on genome-level relationships. For bacterial viruses, a roadmap and guidance for genomic classification have been published with recommendations for analysis of the genomes of these diverse and ubiquitous viruses [2, 29, 30]. The abolishment of the order *Caudovirales* and the families *Myoviridae*, *Siphoviridae*, and *Podoviridae* has resulted in a significant number of floating subfamilies and genera. To resolve this, concerted efforts by the BVS and the wider community are required to establish new taxa at the ranks of family and order that accurately reflect the evolutionary relationships of these viruses. With more research, greater coverage of viral diversity, and the development of new and more-sensitive approaches for sequence analysis, we are confident that robust consensus approaches for the delineation of higher taxa will emerge.

The creation of the order *Crassvirales* and nine families of viruses infecting members of the bacterial class Flavobacteriia serves to illustrate the significance of virome datasets for establishing demarcation criteria for taxonomic classification. The inclusion of viruses identified from metagenomic data is essential to capture the true diversity of bacterial viruses and will undoubtedly lead to the expansion of existing taxa and the creation of new ones in the future. However, for inclusion within the taxonomic framework, it is necessary to incorporate appropriate checks to demonstrate that such sequences represent the complete coding region of the genome [31].

The classification of bacterial viruses unquestionably relies upon the combined efforts of the research community, and the past year has seen increasing numbers of proposals submitted from outside the BVS and its study groups. We strongly encourage such initiatives. Interested researchers can always contact the BVS and study groups for information, consultation, and engagement with the taxonomic framework. While metagenomic virus discovery efforts continue to uncover and catalogue the diversity of the virosphere, taxonomic classification will inevitably lag behind the submission of new genome sequences to databases. We hope that through the engagement of the wider community and the continued development of new tools and approaches for sequence analysis, this gap will decrease substantially in the near future.

## Electronic Supplementary Material

Below is the link to the electronic supplementary material

Supplementary Material 1
